# Environmental prospecting of black yeast-like agents of human disease using culture-independent methodology

**DOI:** 10.1038/s41598-020-70915-0

**Published:** 2020-08-26

**Authors:** Flávia de Fátima Costa, Nickolas Menezes da Silva, Morgana Ferreira Voidaleski, Vinicius Almir Weiss, Leandro Ferreira Moreno, Gabriela Xavier Schneider, Mohammad J. Najafzadeh, Jiufeng Sun, Renata Rodrigues Gomes, Roberto Tadeu Raittz, Mauro Antonio Alves Castro, Graciela Bolzón Inez de Muniz, G. Sybren de Hoog, Vania Aparecida Vicente

**Affiliations:** 1 Engineering Bioprocess and Biotechnology Post-Graduation Program, Department of Bioprocess Engineering and Biotechnology, Federal University of Paraná, Curitiba, Brazil; 2 Microbiology, Parasitology and Pathology Post-Graduation Program, Department of Pathology, Federal University of Paraná, Curitiba, Brazil; 3 Department of Parasitology and Mycology, School of Medicine, Mashhad University of Medical Sciences, Mashhad, Iran; 4 Guangdong Provincial Institute of Public Health, Guangdong Provincial Center for Disease Control and Prevention, Guangzhou, China; 5 Laboratory of Bioinformatics, Professional and Technological Education Sector, Federal University of Paraná, Curitiba, Brazil; 6 Central Laboratory in Nanotechnology, Federal University of Paraná, Curitiba, Brazil; 7 Center of Expertise in Mycology of Radboud University Medical Center/Canisius Wilhelmina Hospital, Nijmegen, The Netherlands

**Keywords:** Classification and taxonomy, Skin diseases, Next-generation sequencing, Bioinformatics, Data mining, Genetic databases, Fungal ecology, Pathogens

## Abstract

Melanized fungi and black yeasts in the family Herpotrichiellaceae (order Chaetothyriales) are important agents of human and animal infectious diseases such as chromoblastomycosis and phaeohyphomycosis. The oligotrophic nature of these fungi enables them to survive in adverse environments where common saprobes are absent. Due to their slow growth, they lose competition with common saprobes, and therefore isolation studies yielded low frequencies of clinically relevant species in environmental habitats from which humans are thought to be infected. This problem can be solved with metagenomic techniques which allow recognition of microorganisms independent from culture. The present study aimed to identify species of the family Herpotrichiellaceae that are known to occur in Brazil by the use of molecular markers to screen public environmental metagenomic datasets from Brazil available in the Sequence Read Archive (SRA). Species characterization was performed with the BLAST comparison of previously described barcodes and padlock probe sequences. A total of 18,329 sequences was collected comprising the genera *Cladophialophora*, *Exophiala*, *Fonsecaea*, *Rhinocladiella* and *Veronaea*, with a focus on species related to the chromoblastomycosis. The data obtained in this study demonstrated presence of these opportunists in the investigated datasets. The used techniques contribute to our understanding of environmental occurrence and epidemiology of black fungi.

## Introduction

A large number of species of black yeast-like fungi that belong to the ascomycetous order Chaetothyriales in the family Herpotrichiellaceae are renowned as opportunistic pathogens in immunocompetent vertebrate hosts^1,2^. Agents are particularly involved in subcutaneous, and systemic or disseminated infections, known as chromoblastomycosis and phaeohyphomycosis, respectively^2–4^. These infections are invariably chronic and can be severely mutilating or even fatal.

Chromoblastomycosis is a relatively common disease in rural tropical climate zones around the world. This implantation disorder is characterized by the presence of a specialized tissue form of the fungus known as the muriform cell^2,5,6^. Infection is hypothesized to take place via traumatic inoculation of environmental material such as plant thorns and/or wood fragments^7,8^. Epidemiological data confirmed by studies using selective isolation methods^9–12^ suggest an environmental origin of this disease. However, presence of these agents is infrequent. Only few isolates have been recovered even after extensive sampling in endemic areas^9, 10,13,14^, where cultures usually only yield non-pathogenic relatives. Novel molecular methods are required for understanding the ecology and environmental occurrence of these agents.

Metagenomics are culture-independent methods for the study of microbial diversity, based on next generation sequencing (NGS) and allowing characterization of fungi in complex environmental systems, using specific molecular markers for identification^15^. Abundant metagenomic data are available in public databases such as Sequence Read Archive (SRA^16^), Rast Server (MG-RAST^17^), and EBI metagenomics (EMG^18^). Likewise, sequences of several molecular markers are available that are in use for taxonomy and routine molecular identification of species in Herpotrichiellaceae, i.e. ITS, *TEF1*, *BT2*, and *ACT1*^19^. Alternatively, padlock probes, which are specific oligonucleotides with the ability to identify single nucleotide polymorphisms (SNPs), have been proposed for the recognition of several groups of black agents^20–25^. DNA barcoding, based on the ITS region and applying short sequences (25‒41 bp) of nucleotides specific for a single taxonomic species^26^, can additionally be used to recognize herpotrichiellaceous species by variable regions in the ribosomal operon.

The present study aims to explore the environmental occurrence of chromoblastomycosis agents in the family Herpotrichiellaceae in environmental samples in tropical areas of Brazil. We compare metagenomic data present in public databases, using barcodes and padlock probes for species identification. This approach should lead to better understanding of the sources and routes of infection of patients with chromoblastomycosis.

## Results

### Datasets containing herpotrichiellaceous fungi

**Figure 1:**
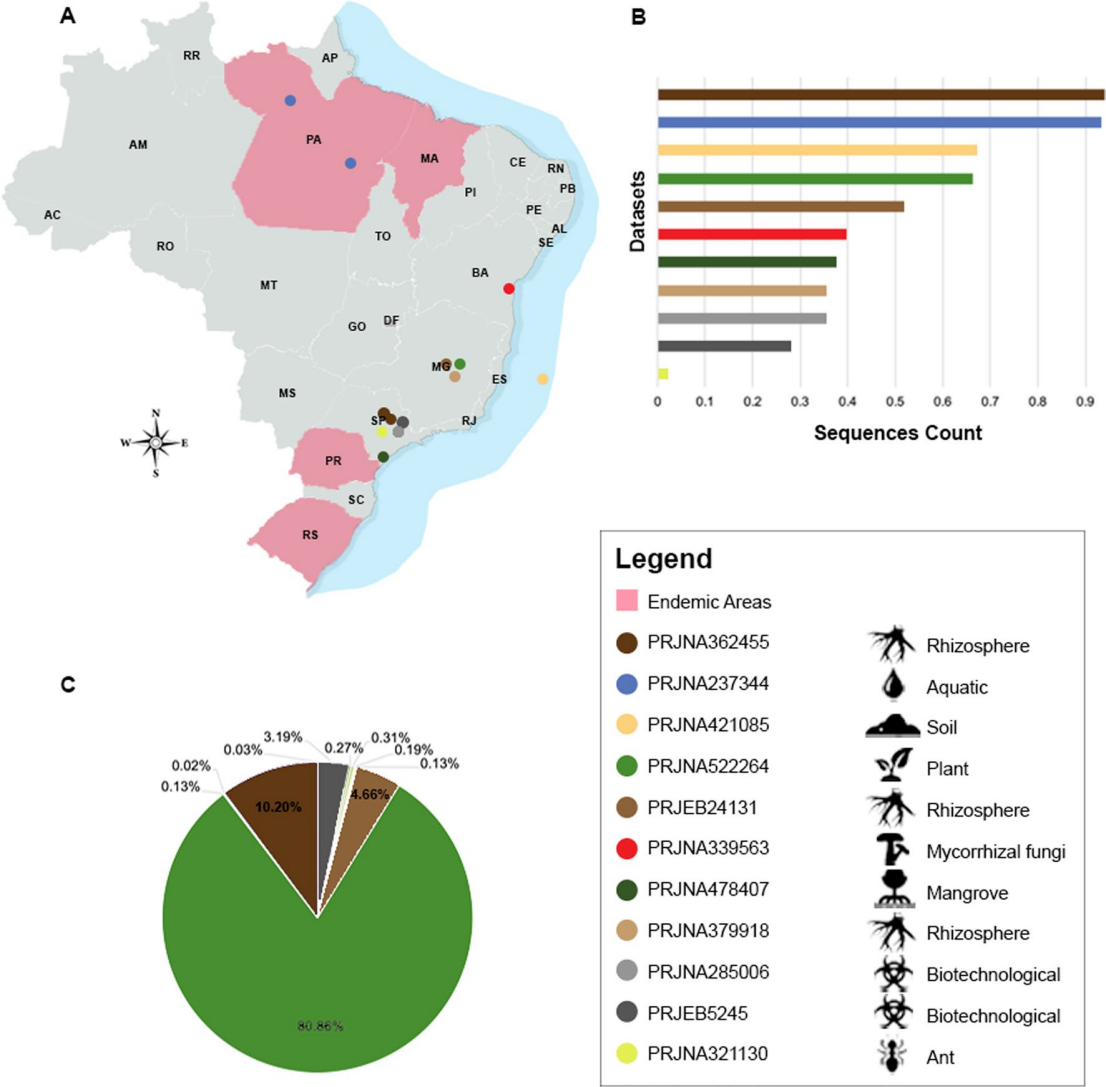
Herpotrichiellaceous sequences encountered in investigated datasets. (**A**) Geographic metagenomic data distribution. (**B**) Total of reads in investigated datasets. (**C**) Herpotrichellaceous sequences per dataset. The image was created using Adobe Photoshop CC (v. 20.0.4) based on the map (https://commons.wikimedia.org/wiki/File:20111110231441!Estados_de_nascimento_de_presidentes_brasileiros.png), which is available under a Creative Commons license https://creativecommons.org/licenses/by-sa/3.0/deed.en.

In total, 169 large datasets distributed in 3,786 samples from Brazil were analyzed (Table S1). Of these, only 11 large datasets arranged in 179 samples have sequences of members of Herpotrichiellaceae, originating from five states and representing environmental samples from different geographic areas (Fig. 1A).

The generated data was according to the scope of each metagenome project evaluated, which resulted in a high variation in size of the datasets. The read number ranged from 14,293 to 1,394,769,476, with the rhizosphere metadata (PRJNA362455) being the one with the highest number of reads (Table 1; Fig. 1B). Within each read pool, the ones matching Herpotrichiellaceae ranged from 4 reads to 14,821 sequences, with the highest concentration in the plant metadata (PRJNA522264). All results considered normalized data (Table 1; Fig. 1C).

**Table 1:** Overview of sequences identified as fungi in Herpotrichiellaceae.

Source	Accession large datasets	Number of sequences total	Number of sequences of Herpotrichiellaceae identified	Criteria of species identification		
				Barcode^a^	Padlock probes^b^	Both^c^
Rhizosphere	PRJNA379918	2,895,509	57	0	11	46
	PRJNA362455	1,394,769,476	1,870	1,866	0	4
	PRJEB24131	11,210,858	855	0	252	603
Ant	PRJNA321130	142,930	6	6	0	0
Aquatic	PRJNA237344	1,104,240,094	4	2	0	2
Biotechnological	PRJNA285006	2,889,538	50	6	1	43
	PRJEB5245	1,498,794	584	0	565	19
Mycorrhizal	PRJNA339563	4,146,905	24	4	17	3
Plant	PRJNA522264	35,414,582	14,821	13,634	136	1,051
Mangrove	PRJNA478407	3,505,958	35	8	27	0
Soil	PRJNA421085	38,540,232	23	0	23	0
	Total	2,599,126,239	18,329	15,526	1,032	1,771

Identification by only barcodes^a^, only padlock probes^b^, and padlock probes and barcodes simultaneously^c^.

The total number of reads matching herpotrichiellaceous fungi was 18,329. Of this data pool, 84% (15,526 reads) were identified by barcode markers, and only around 5.6% (1,032 reads) exclusively by padlock probe markers. The number of sequences identified simultaneously by both markers were 1,771 reads (Table 1), which underlined the requirement to use more than a single tool for in silico identification.

### Species identified

In the datasets investigated, the genera *Cladophialophora*, *Exophiala*, *Fonsecaea*, *Rhinocladiella* and *Veronaea* were identified. The sequences mainly belonged to the genus *Exophiala,* which was identified by barcodes and padlock probes. Among the fungi from the family Herpotrichiellaceae, *Exophiala* species were the most abundantly represented (18,113 sequences) corresponding to 98.77% of the total sequences belonging to 16 described *Exophiala* species of which *E. bergeri* (46.01%), *E. sideris* (27.86%), and *E. pisciphila* (11.42%) were prevalent. The presence of *Fonsecaea pedrosoi*, the major agent of chromoblastomycosis in Brazil, was detected at low incidence (0.74%). *Cladophialophora* species (0.14%) were represented by *C. chaetospira* (0.12%), and *C. arxii* and *C. immunda*, both with 0.01%. Of the genus *Rhinocladiella* (0.30%), two species were identified, i.e. *R. similis* (0.08%) and *R. atrovirens* (0.22%). *Veronaea botryosa* was present in low numbers (0.05%) (Table 2).

**Table 2:** Species identified in metagenomic datasets from different regions in Brazil.

Species identified in silico	Sources sampling											Total of sequences	%
	PRJNA 379918 Rhizos	PRJNA 362455 Rhizos	PRJEB 24131 Rhizos	PRJNA 321130 Ant	PRJNA 237344 Aq	PRJNA 285006 Biotec	PRJEB 5245 Biotec	PRJNA 339563 Myco	PRJNA 522264 Plant	PRJNA 478407 Mang	PRJNA 421085 Soil		
*Exophiala angulospora*	0	0	0	0	0	0	0	0	0	4^b^	0	4	0.02
*Exophiala bergeri*	0	0	1^a^	0	0	1^a^	0	0	8,431^a^	0	0	8,433	46.01
*Exophiala brunnea*	0	10^a^	2^a^	0	0	0	0	4^a^	1^a^	0	0	17	0.09
*Exophiala cancerae*	0	0	7^a^	0	0	1^a^	562^a^	0	35^a^	0	0	605	3.30
*Exophiala castellanii*	0	0	0	0	0	0	0	3^c^	0	0	0	3	0.02
*Exophiala pisciphila*	10^a^	1,856^a^	200^a^	0	0	0	0	0	0	23^b^	5^b^	2,094	11.42
*Exophiala exophialae*	1^a^	0	0	6^a^	1^a^	0	0	0	0	0	0	8	0.04
*Exophiala equina*	0	0	20^a^	0	0	0	0	0	0	0	0	20	0.11
*Exophiala dermatitidis*	0	0	0	0	2^c^	1^c^	0	0	0	0	0	3	0.02
*Exophiala heteromorpha*	0	0	0	0	1^a^	0	0	0	0	0	0	1	0.01
*Exophiala jeanselmei*	0	0	0	0	0	0	19^c^	0	0	0	0	19	0.10
*Exophiala sideris*	0	0	0	0	0	0	0	0	5,106^a^	0	0	5,106	27.86
*Exophiala spinifera*	0	0	603^c^	0	0	42^c^	0	0	0	0	0	645	3.52
*Exophiala xenobiotica*	46^c^	4^c^	0	0	0	1^b^	0	16^b^	1,051^c^	0	0	1,118	6.10
*Exophiala oligosperma*	0	0	0	0	0	0	3^a^	1^a^	20^a^	0	0	24	0.13
*Exophiala mesophila*	0	0	0	0	0	3^a^	0	0	0	0	0	3	0.02
*Cladophialophora arxii*	0	0	0	0	0	0	0	0	0	1^b^	0	1	0.01
*Cladophialophora chaetospira*	0	0	0	0	0	0	0	0	0	6^b^	16^b^	22	0.12
*Cladophialophora immunda*	0	0	0	0	0	0	0	0	0	0	2^b^	2	0.01
*Fonsecaea pedrosoi*	0	0	0	0	0	0	0	0	136^b^	0	0	136	0.74
*Rhinocladiella atrovirens*	0	0	0	0	0	0	0	0	41^a^	0	0	41	0.22
*Rhinocladiella similis*	0	0	14^a^	0	0	0	0	0	0	0	0	14	0.08
*Veronaea botryosa*	0	0	8^a^	0	0	1^a^	0	0	0	1^b^	0	10	0.05
Total	57	1,870	855	6	4	50	584	24	14,821	35	23	18,329	

*Rhizos.* rhizosphere, *Aq.* aquatic, *Biotec.* sugarcane filter cake and lignocellulosic biomass, *Myco.* mycorrhizal, *Mang.* mangrove.

Identification by only padlock probes^a^, only barcodes^b^, and padlock probes and barcodes simultaneously^c^.

Among the datasets analyzed, the largest number of species was found in soil-associated material and in plants of the family Velloziaceae (PRJNA522264) and in root-associated debris of maize (PRJEB24131), i.e. *E. sideris*, *E. xenobiotica*, *E. oligosperma*, *F. pedrosoi* and *R. atrovirens* which were found only in the first dataset, and *E. spinifera*, *E. pisciphila*, *E. equina*, *R. simili*s and *V. botryosa* in second dataset, while *E. bergeri, E. brunnea* and *E. cancerae* were present in both sources. In maize rhizosphere (PRJNA379918) and citrus rhizosphere (PRJNA362455) *E. pisciphila* and *E. xenobiotica* were found, while *E. brunnea* was present only in the citrus source and *E. exophialae* in maize. In mycorrhizal fungi (PRJNA339563), *E. castellanii*, *E. oligosperma*, *E. brunnea*. *E. xenobiotica* were identified. In mangrove (PRJNA478407), *E. angulospora*, *E. pisciphila*, *C. arxii*, *C. chaetospira* and *V. botryosa* were present. In lignocellulosic biomass (PRJEB5245), *E. jeanselmei* and *E. cancerae* were identified. In soils contaminated with crude oil (PRJNA421085), *E. pisciphila*, *C. immunda* and *C. chaetospira* were present. Moreover, in the sugarcane filter cake (PRJNA285006), *E. dermatitidis*, *E. mesophila*, *E. spinifera*, *E. bergeri*, *E. cancerae*, *E. xenobiotica* and *V. botryosa* were identified. The river water source (PRJNA237344) showed sequences of *E. heteromorpha*, *E. dermatitidis* and *E. exophialae*. The dataset with sequences associated with ants (PRJNA321130) identified only *E. exophialae* (Table 2).

## Discussion

In this study we investigated the presence of sequences of herpotrichiellaceous fungi in metagenomic datasets that were generated after analysis of divergent environmental sources, using molecular markers for in silico identification of causal agents of chromoblastomycosis and phaeohyphomycosis. The tools used as reference were padlock probes developed for rapid detection of pathogenic *Fonsecaea* species in clinical samples (*F. pedrosoi*, *F. nubica*, *F. monophora* and *F. pugnacius*^20,24^), the agent of neurotropic phaeohyphomycosis *Cladophialophora bantiana*^23^, and other opportunistic species with variable pathology^21,22,25^. ITS rDNA barcoding sequences had previously been recommended for rapid identification of clinical and environmental sequences^27^, and were suggested for taxonomic identification in metagenomic data^26^.

The results indicated that this methodology represents complementary data to studies on direct isolation via culture^9–14,28^, which all reported low frequency of these agents in the environment. Judging from the number of sequences present in the evaluated datasets, the low frequency of herpotrichiellaceous fungi, compared to the total number of fungal sequences, was confirmed (Table 1). For example, *Fonsecaea pedrosoi,* a major agent of chromoblastomycosis in Brazil^2^, was detected in metagenomic data from plant- and soil-associated materials. This habitat is in line with the hypothesis of chromoblastomycosis as an implantation disease from inoculated plant-derived material. This demonstrates that in silico identification can be used as a new tool to uncover the natural habitat of agents of opportunistic diseases and assists in elucidating the environmental occurrence and the route of infection of causative species.

The infection route of agents of chromoblastomycosis nevertheless remains controversial. Their occurrence in living plants has extensively been discussed. Previous studies have shown that *Fonsecaea* species occurring in living plant material mostly belong to other species than those repeatedly encountered on the human host^13,29^. In our study, the non-pathogenic *Fonsecaea* species were not detected. A study presented an in vitro plant infection model showing that the agents of human chromoblastomycosis have a certain degree of plant-invasive ability^30^, suggesting that those species occur on plants as well. We may hypothesize, that both strictly saprobic and opportunistic species are very rare and thus both have a low chance to be detected in non-optimal datasets using unbiased methodology. Differences in habitat choice, even when minute, may influence species-specific population dynamics and representation in metagenomics datasets, slight differences determining presence or absence.

Species of the genus *Rhinocladiella* have been described as less common agents of chromoblastomycosis^31,32^, i.e. *R. aquaspersa, R. similis* and *R. tropicalis*^3^. The extremely rare agent *Rhinocladiella similis* has also been isolated from dialysis water and from babassu coconuts^14,33^, while in our in silico data, *R. similis* was observed in the rhizosphere of maize. The human host thus is unlikely to be the prime habitat of *R. similis*. The saprobe *R. atrovirens* was identified in plant and soil-associated habitats. In addition, *Veronaea botryosa,* an extremely rare agent of disseminated infections in patients with *CARD9* immune disorders^34,35^, had previously been isolated from babassu coconuts^14^ and from creosote-treated railway ties^10^. In this study, the species was identified in mangrove, maize rhizosphere and in sugarcane filter cake, indicating a wider saprobic occurrence.

Presence of herpotrichiellaceous opportunists in the environment has been shown by several authors^8–14,28^. Our in silico data showed that the most common sequences in metagenomic databases belonged to the genus *Exophiala*. This is the largest genus in the family Herpotrichiellaceae containing numerous species, many of which are opportunistic pathogens of cold- and warm-blooded animals^19,36^. We detected species reported from various types of disease other than chromoblastomycosis, i.e. *E. bergeri*, *E. dermatitidis*, *E. jeanselmei*, *E. heteromorpha*, *E. mesophila*, *E. spinifera*, *E. oligosperma* and *E. xenobiotica*^37^. Also *E. angulospora*, *E. pisciphila* and *E. equina,* associated with infections of cold-blooded animal such as frogs, toads and fish^36,38^ were detected. *Exophiala cancerae* was first described from the Lethargic crab disease (LCD) occurring along the Brazilian coas^36,39^. This species hitherto had only been found in endemic coastal areas. However, in our study it was identified in soil, plant roots and in a sugar filter cake, indicating a wider environmental occurrence. Other unexpected encounters were *E. castellanii,* previously isolated from water^40^ but in our data among mycorrhizal fungi, *E. brunnea,* known from litter^36^ but here in association with mycorrhizal fungi, rhizosphere and plant, and *E. sideris* from the hydrocarbon-polluted environments^41^ but here from plant- and soil-associated materials, and finally *E. exophialae* known from straw in a burrow of *Dasypus septemcinctus*, but here from river water, rhizosphere and associated with ants.

The genus *Cladophialophora* was represented by two opportunistic species, *C. arxii* and *C. immunda*. *Cladophialophora arxii* was originally reported from a disseminated infection^42^ and *C. immunda* from a patient with a subcutaneous ulcer^43^. The latter species was later detected in sites polluted with hydrocarbons^44^, which matches with its presence in soils contaminated with crude oil analyzed in this study. The environmental saprobe *C. chaetospira* is known to occur in plant litter^10, 43^, while in our study it was found in mangroves and in soil contaminated with crude oil.

## Conclusions

The methodology presented in this study was shown to be a reliable and quick alternative to identify the presence of agents of clinical interest in environmental samples, which is particularly valid for fungi that are difficult to bring in culture, such as black yeasts and other opportunistic agents of human disease. The use of molecular markers as tools for the identification of Herpotrichiellaceae in metagenomic datasets proved to be an effective way to study microhabitats of these fungi, demonstrating the importance of mining databanks for tracking fungal agents. Although local, Brazilian databases were used, the investigated fungi have global distributions, and results are likely to be similar elsewhere. However, data availability is still limited, since the barcode sequences and padlocks described in the literature are restricted to relatively few species. This may explain why in a number of cases our data are significantly different from existing literature, in that common saprobic relatives were not detected, while species with supposedly limited distribution were found in remote, variable habitats suggesting a low degree of host- or habitat-specificity. Expansion of databases may provide a more balanced picture in the future.

## Materials and methods

### Database construction

The metagenomic database was created based on projects disponible in the Sequence Read Archive (SRA) (https://www.ncbi.nlm.nih.gov/sra). To search the projects, the term “metagenomic Brazil” was used and all projects were downloaded. This dataset contained a total of 3,786 samples with approximately 2 terabytes (Table S1). The database was assembled only with metagenomes that complied with four criteria: (1) DNA sequences; (2) Brazilian projects to narrow down the selection; (3) environmental link (arthropods and other animals, aquatic bodies, hostile environments including rocks, decomposing materials with plant debris and soil), since within the geographic area the actual habitat is unknown; (4) public data available for download in the SRA. The datasets were rearranged according to eight types of sources, i.e. rhizosphere (PRJNA379918, PRJNA362455, PRJEB24131), ant (PRJNA321130), aquatic (PRJNA237344), biotechnological (PRJNA285006, PRJEB5245), mycorrhizal (PRJNA339563), plant (PRJNA522264), mangrove (PRJNA478407), and soil (PRJNA421085) (Table 3).

**Table 3:** Summary of selected datasets that contain sequences of fungi in Herpotrichiellaceae.

Accession large datasets	Accession samples	Dataset description
PRJNA379918	SRR5399784, SRR5399785, SRR5399787, SRR5399789	Rhizosphere: maize rhizosphere community under different phosphate sources^45^^a^
PRJNA362455	SRR5195137, SRR5195141, SRR8056346, SRR8056347, SRR8056355 to SRR8056358	Rhizosphere: citrus rhizosphere microbiome^46^
PRJEB24131	ERR2233399 to ERR2233446	Rhizosphere: root-associated microbiome of maize genotypes with contrasting phosphorus use efficiency^47^^a^
PRJNA321130	SRR3493327	Ant: the fungal diversity found on the integument of *Atta capiguara* and *A. laevigata alate* ants^48^
PRJNA237344	SRR1786616, SRR1790680, SRR4833059	Aquatic: evaluation of the waters of the Amazon River to the Atlantic Ocean, with sensitivity to climate variability and anthropogenic forces due to their immense scale: the Amazon River-Ocean Continuum (https://amazoncontinuum.org/)
PRJNA285006	SRR2086459, SRR2086461, SRR2086464, SRR2086481	Biotechnological: microbiome sugarcane filter cake compost piles to analyse the dynamics of fungal and bacterial communities along the process and biomass degrading profile for second generation bioethanol: CNPEM (https://cnpem.br/)
PRJEB5245	ERR957350, ERR957352 to ERR957355	Biotechnological: development of a microbial enrichment for sugarcane bagasse breakdown: CNPEM (https://cnpem.br/)
PRJNA339563	SRR4065317, SRR4065319, SRR4065500	Mycorrhizal: interactions of tropical mycoheterotrophic plants and their arbuscular mycorrhizal fungal hosts^49^
PRJNA522264	SRR8585376, SRR8585377, SRR8585380, SRR8585381, SRR8585384 to SRR8585386, SRR8585391, SRR8585392, SRR8585395 to SRR8585398, SRR8585411, SRR8585412, SRR8585414, SRR8585416, SRR8585417, SRR8585420, SRR8585425, SRR8585428, SRR8585430, SRR8585434 to SRR8585437, SRR8585453 to SRR8585457, SRR8585459, SRR8585461 to SRR8585465, SRR8585467, SRR8585468, SRR8585471, SRR8585474, SRR8585475, SRR8585489, SRR8585492, SRR8585494 to SRR8585497, SRR8585501 to SRR8585503, SRR8585506, SRR8585507, SRR8585509, SRR8585510, SRR8585513 to SRR8585518, SRR8585520, SRR8585530 to SRR8585533, SRR8585536, SRR8585538	Plant: characterization of the microbiomes associated with two plant species (*Vellozia epidendroides* and *Barbacenia macrantha*) that thrive in the extremely P-impoverished soils of the Brazilian *campos rupestres*: the Genomics for Climate Change Research Center (https://www.genclima.cnptia.embrapa.br/)
PRJNA478407	SRR7450155 to SRR7450157, SRR7450161 to SRR7450169, SRR7450174, SRR7450176, SRR7450177, SRR7450179, SRR7450181, SRR7450186	Mangrove: metagenomics and metatranscriptomics of the microbial community involved in the transformation of organic carbon in mangrove sediments at São Paulo state: Embrapa Environment (https://www.embrapa.br/meio-ambiente)
PRJNA421085	SRR6354886	Soil: soils contaminated with crude oil^50^

^a^Different projects with related aims.

### Identification tools

The molecular markers for members of the family Herpotrichiellaceae described in the literature (Table S2) were used for species identification in the metagenome datasets. A total of 97 barcode identifiers with 25‒41 bp^26^ and 25 padlock probes sequences with 28‒42 bp with different SNPs were collected from an rDNA internal transcribed spacer (ITS2^20–25^).

### Identification in silico

**Figure 2:**
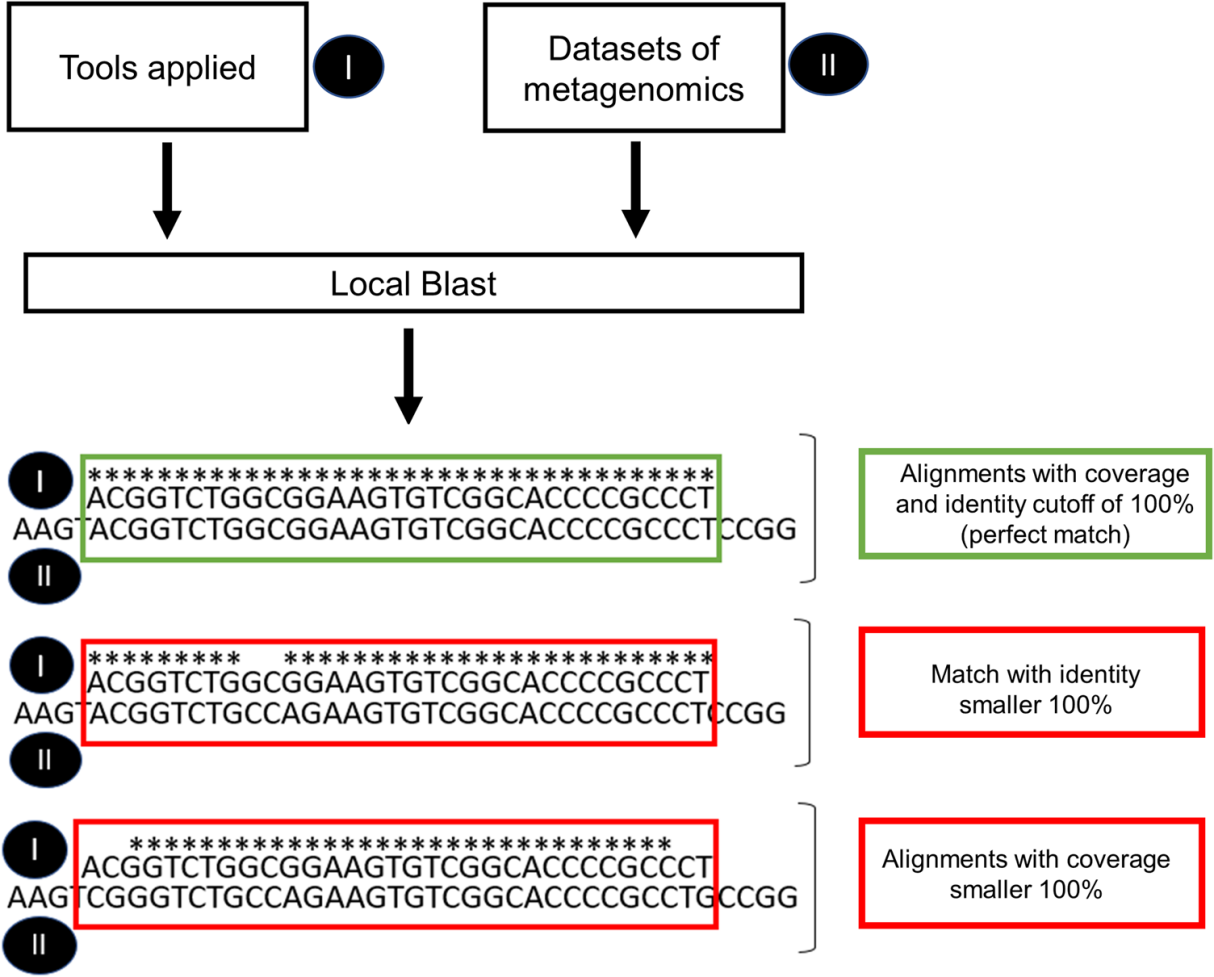
Fluxogram of identification in silico. In green criteria of selection and in red rejected criteria.

Comparison of metagenomes with molecular marker sequences was performed with local BLASTn (v2.6.0.+). For the data mining, only alignments with coverage and identity cutoff of 100% (perfect match) were considered (Fig. 2). Matches with values below the cutoff were excluded. Because padlock and barcode probes are extremely specific for species identification, cases of slight misalignment and non-perfect sequence identity do not characterize the fungus in the analyses (Fig. 2). Metagenome reads from double-strand sequencing where considered once in the final read count.

## Supplementary information

Supplementary Table S1.

Supplementary Table S2.
