# Supplementary material for: Environmental prospecting of black yeast-like agents of human disease using culture-independent methodology
Source: Sci Rep. 2020 Aug 26;10:14229. doi: 10.1038/s41598-020-70915-0 (PMC7450056; doi:10.1038/s41598-020-70915-0)
Supplement: Supplementary file 2 — Supplementary Table S2. [file 41598_2020_70915_MOESM2_ESM.pdf]

Environmental prospecting of black yeast-like agents of human disease using culture-independent methodology

Flávia de Fátima Costa[1], Nickolas Menezes da Silva[1], Morgana Ferreira Voidaleski[2], Vinicius Almir Weiss[2], Leandro Ferreira Moreno[2], Gabriela Xavier Schneider[2], Mohammad J. Najafzadeh[3], Jiufeng Sun[4], Renata Rodrigues Gomes[2], Roberto Tadeu Raittz[5], Mauro Antonio Alves Castro[5], Graciela Bolzón Inez de Muniz[6], G. Sybren de Hoog[2,7,*], Vania Aparecida Vicente[1,2,*]

[1] Engineering Bioprocess and Biotechnology Post-Graduation Program, Department of Bioprocess Engineering and Biotechnology, Federal University of Paraná, Curitiba, Brazil

[2] Microbiology, Parasitology and Pathology Post-Graduation Program, Department of Pathology, Federal University of Paraná, Curitiba, Brazil

[3] Department of Parasitology and Mycology, School of Medicine, Mashhad University of Medical Sciences, Mashhad, Iran

[4] Guangdong Provincial Institute of Public Health, Guangdong Provincial Center for Disease Control and Prevention, Guangzhou, China

[5] Laboratory of Bioinformatics, Professional and Technological Education Sector, Federal University of Paraná, Curitiba, Brazil

[6] Central Laboratory in Nanotechnology, Federal University of Paraná, Curitiba, Brazil

[7] Center of Expertise in Mycology of Radboud University Medical Center / Canisius Wilhelmina Hospital, Nijmegen, The Netherlands

Supplementary Tables

Supplementary Table S2. Molecular markers described in the literature of family Herpotrichiellaceae.

Species	Strains	Source	Number pb		Ref.
			Barcodes	Probes padlock	
Cladophialophora arxii	CBS 306.94T	Human	35	42	[1, 2]
	CBS 409.96	Human	37	-	[1]
Cladophialophora bantiana	CBS 173.52	Human	34	41	[1, 2]
Cladophialophora chaetospira	CBS 115468	Plant	-	39	[2]
Cladophialophora carrionii	CBS 160.54LT	Human	29	42 /39	[1-3]
	CBS 163.54	Human	29	-	[1]
	CBS 260.83	Human	29	-	[1]
	FMC.248	Human	29	-	[1]
Cladophialophora devriesii	CBS 147.84T	Human	25	42	[1, 2]
	IFM51369	-	25	-	[1]
Cladophialophora emmonsii	CBS 979.96T	Human	33	-	[1]
	CBS 640.96	Cat, subcutaneos lesion	33	-	[1]
Cladophialophora immunda	CBS 834.96T	Human	25	41 / 42	[1,2]
	CBS 102227	Plant	25	-	[1]
Cladophialophora minourae	CBS 987.96	Rotting wood	-	42	[2]
Cladophialophora modesta	CBS 985.96T	Human	26	-	[1]
Cladophialophora mycetomatis	CBS 122637T	Human	28	-	[1]
	CBS 454.82	Culture contaminant	28	-	[1]
Cladophialophora psammophila	CBS 110553T	Gasoline-polluted soil	34	41	[1, 2]
Cladophialophora samoensis	CBS 259.83T	Human	31	-	[1]
Cladophialophora saturnica	CBS 118724T	Child	27	42	[1,2]
Cladophialophora subtilis	CBS 12264T	Iced tea	30	-	[1]
Cladophialophora yegresii	CBS 114405T	Plant	30	42	[1,2]
Exophiala alcalophila	CBS 520.82T	Soil	31	-	[1]
Exophiala angulospora	CBS 482.92T	Water	28	36	[1, 4]
	CBS 122264	Hydrocarbon-polluted soil	28	-	[1]
	CBS 109906	Water	28	-	[1]
Exophiala aquamarina	CBS 119918T	Seadragon, skin	38	37	[1, 4]
Exophiala asiatica	BMU00015T	Human	36	-	[1]
Exophiala attenuata	CBS110026	Human	29	-	[1]
Exophiala bergeri	CBS 353.52T	Human	31	-	[1]
Exophiala brunnea	CBS 587.66T	Plant	38	-	[1]
Exophiala cancerae	CBS 120532T	Crab	34	-	[1]
Exophiala castellanii	CBS 158.58T	Human	28	37	[1,4]

Exophiala capensis	CBS 128771^{T}	Plant	31	-	[1]
Exophiala dermatitidis	CBS 207.35^{T}	Human	32	36	[1, 5]
	CBS 100338	Humidifier	32	-	[1]
	BMU00035	Human	32	-	[1]
Exophiala equina	CBS 119.23^{T}	Horse skin	38	-	[1]
	CBS 122263	Human foot	38	-	[1]
	CBS 122270	Human foot	38	-	[1]
Exophiala exophialae	CBS 668.76^{T}	Straw in burrow	32	-	[1]
Exophiala halophila	CBS 121512^{T}	Human	34	-	[1]
Exophiala heteromorpha	CBS 232.33^{T}	Wood pulp	29	-	[1]
Exophiala jeanselmei	CBS 507.90^{T}	Human	36	37	[1, 5]
	CBS 677.76	Human	37	-	[1]
Exophiala lacus	CBS 117497^{T}	Water	37	-	[1]
Exophiala lecanii-corni	CBS 123.33^{T}	Human	29	-	[1]
Exophiala mesophila	CBS 402.95^{T}	Shower	28	-	[1]
	CBS 121511	Human	28	-	[1]
Exophiala moniliae	CBS 520.76^{T}	Plant	26	-	[1]
Exophiala nishimurae	CBS 101538^{T}	Bark	32	-	[1]
Exophiala oligosperma	CBS 725.88^{T}	Human	38	37	[1, 5]
	UTHSC95-2041	Human	38	-	[1]
	UTHSC91###870	Human	39	-	[1]
	IFM41701	Soil	40	-	[1]
Exophiala opportunistica	CBS109811	Water	39	36	[1,4]
Exophiala pisciphila	CBS 537.73^{T}	Fish	39	37	[1, 4]
Exophiala salmonis	CBS 157.67^{T}	Fish	37	-	[1]
Exophiala sideris	CBS 121818^{T}	Plant	29	-	[1]
Exophiala spinifera	CBS 899.68^{T}	Human	32	34	[1,5]
Exophiala siphonis	UTHSC88-471	Human	27	-	[1]
Exophiala xenobiotica	CBS 118157^{T}	Oil sludge	30	37	[1, 5]
	CBS 119306	Human	30	-	[1]
	CBS 117665	Human	30	-	[1]
	CBS 117676	Human	33	-	[1]
	CBS 117641	Human	32	-	[1]
Fonsecaea erecta	dH20513	Plant	33	-	[1]
	dH20502	Plant	33	-	[1]
Fonsecaea minima	dH20511	Plant	33	-	[1]
Fonsecaea monophora	CBS 269.37^{T}	Human	36	28	[1, 6]
	CBS 121732	Human	36	-	[1]
	IFM4889	Human	36	-	[1]
	IFM54446	Human	36	-	[1]

Fonsecaea multimorphosa	CBS 980.96^T	Cat	27	-	[1]
Fonsecaea nubica	CBS 269.64^T	Human	36	36	[1, 6]
	CBS 121733	Human	36	-	[1]
	CBS 557.76	Unknown	36	-	[1]
Fonsecaea pedrosoi	CBS 271.37^T	Human	36	36	[1, 6]
	CBS 122741	Human	37	-	[1]
Fonsecaea pugnacius	CBS 139214	Human	-	31	[7]
Phialophora americana	CBS 840.69	Decaying timber	30	-	[1]
Phialophora verrucosa	CBS 273.37	Human	30	-	[1]
	IMTSP.800	Soil	30	-	[1]
	CBS 286.47	Plant	30	-	[1]
	CBS 839.68	Kiwifruit elephantiasis	30	-	[1]
Rhinocladiella anceps	CBS 181.65	Soil	30	-	[1]
	CBS 157.54	Plant	32	-	[1]
Rhinocladiella aquaspersa	CBS 313.73^T	Human	27	-	[1]
Rhinocladiella atrovirens	CBS 264.49	Honey	28	-	[1]
Rhinocladiella basitona	CBS 101460^T	Human	31	-	[1]
Rhinocladiella mackenziei	CBS 650.93^T	Human	37	-	[1]
	CBS 367.92	Human	35	-	[1]
	CBS 102590	Human	35	-	[1]
Rhinocladiella similis	CBS 111763^T	Human	32	-	[1]
	dH13054	Water	32	-	[1]
Veronaea botryosa	CBS 254.57^T	Sansa olive slag	40	35	[1, 4]
	CBS 350.65	Dung of goat	41	-	[1]
Veronaea compacta	CBS 268.75^T	Unknown	38	-	[1]
Veronaea japonica	CBS 776.83^T	Dead bamboo culm	38	-	[1]

References

1. Heinrichs, G., De Hoog, G. S. & Haase, G. Barcode identifiers as a practical tool for reliable species assignment of medically important black yeast species. *J. Clin. Microbiol.* **50**, 3023–3030, 10.1128/JCM.00574-12 (2012).
2. Hamzehei, H. *et al.* Use of rolling circle amplification to rapidly identify species of *Cladophialophora* potentially causing human infection. *Mycopathologia.* **175**, 431–438, 10.1007/s11046-013-9630-7 (2013).
3. Deng, S. *et al.* Three isothermal amplification techniques for rapid identification of *Cladophialophora carrionii*, an agent of human chromoblastomycosis. *J. Clin. Microbiol.* **52**, 3531–3535, 10.1128/JCM.01033-14 (2014).
4. Najafzadeh, M. J. *et al.* Rapid identification of seven waterborne Exophiala species by RCA DNA padlock probes. *Mycopathologia.* **183**, 669–677, 10.1007/s11046-018-0256-7 (2018).
5. Najafzadeh, M. J. *et al.* Detection and identification of opportunistic *Exophiala* species using the rolling circle amplification of ribosomal internal transcribed spacers. *J. Microbiol. Methods.* **94**, 338–342, 10.1016/j.mimet.2013.06.026 (2013).
6. Najafzadeh, M. J., Sun, J., Vicente, V. A. & de Hoog, G. S. Rapid identification of fungal

pathogens by rolling circle amplification using *Fonsecaea* as a model. *Mycoses*. **54**, 577–582, 10.1111/j.1439-0507.2010.01995.x (2011).

7. Schneider, G. X. *et al.* New molecular markers distinguishing *Fonsecaea* agents of chromoblastomycosis. *Mycopathologia.* **184**, 493–504, 10.1007/s11046-019-00359-2 (2019).
